# Odor lateralization and spatial localization: Null effects of blindness

**DOI:** 10.3758/s13414-019-01717-4

**Published:** 2019-04-16

**Authors:** Agnieszka Sorokowska, Anna Oleszkiewicz, Michał Stefańczyk, Justyna Płachetka, Olga Dudojć, Krzysztof Ziembik, Dominika Chabin, Thomas Hummel

**Affiliations:** 1 Smell and Taste Research Lab, Institute of Psychology, University of Wroclaw, ul. Dawida 1, 50-527 Wroclaw, Poland; 2 Smell and Taste Clinic, Department of Otorhinolaryngology, TU Dresden, Dresden, Germany

**Keywords:** Blindness, Visual impairment, Olfaction, Lateralization, Localization, Sensory compensation

## Abstract

People can navigate through an environment using different sensory information, including olfactory cues. Correct intranasal localization and external location of odors can be learned, and some people are able to lateralize olfactory stimuli above chance, which raises the question: What determines the spectrum of olfactory localization abilities. Here, we explored whether odor lateralization and localization abilities are increased in the course of sensory compensation. In a series of studies, we combined two different aspects of odor localization. Study 1 compared abilities of 69 blind people (*M*_age_ = 41 ± 1.6 years; 32 females) and 45 sighted controls (*M*_age_ = 38.3 ± 2.1 years; 25 females) to correctly lateralize eucalyptol, an odorant with a strong trigeminal component, presented to either nostril. Studies 2 and 3 involved a more ecologically valid task, namely spatial localization of olfactory stimuli. In Study 2, 13 blind individuals (*M*_age_ = 28.5 ± 3.5 years; seven females) and 16 sighted controls (*M*_age_ = 34.9 ± 3.2 years; ten females) tried to localize a single odorant, while in Study 3, 97 blind individuals (*M*_age_ = 43.1 ± .5 years; 48 females) and 47 sighted controls (*M*_age_ = 38.7 ± .7 years; 27 females) attempted to localize a single target odor in an experimental olfactory space comprising four different odorants. Blind and sighted subjects did not differ in their abilities to lateralize and to localize odors, and their performance across all tasks suggests that odor lateralization and localization are important for navigation in an environment regardless of visual status.

## Introduction

People can navigate through an environment using different sensory information. Although for senses like vision and audition it is obvious that bilateral perception contributes to accurate spatial orientation, the functions of bilateral olfactory perception are not fully clear. Von Békésy (1964) suggested that time delay and internostril intensity differences can help people determine the location of an odor source. A higher concentration of an odorant would then indicate shorter distance from the odor source. In this case, bilateral olfactory cues would contribute to accurate spatial orientation and localization.

Studies investigating human ability to localize odors intranasally suggest that lateralization (i.e., correct identification of the nostril receiving olfactory stimulation) of odorants activating exclusively the olfactory receptors is difficult. While a few studies showed that people are able to lateralize phenyl ethyl alcohol (PEA) (Kobal & Hummel, 1992; Porter, Anand, Johnson, Khan, & Sobel, 2005), most studies yield opposite results (Frasnelli, Charbonneau, Collignon, & Lepore, 2009; Frasnelli, La Buissonnière Ariza, Collignon, & Lepore, 2010; Moessnang, Finkelmeyer, Vossen, Schneider, & Habel, 2011; Radil & Wysocki, 1998; Wysocki, Cowart, & Radil, 2003). Thus, it seems that selective activation of the olfactory system is not enough for people to lateralize odors correctly – both olfactory and trigeminal receptors of the nasal mucosa need to be involved in order to increase perception accuracy (Kleemann et al., 2009). Consequently, stimuli with a “trigeminal component” were quite consistently found to be lateralized correctly (Frasnelli et al., 2010; Hummel, Futschik, Frasnelli, & Hüttenbrink, 2003; Kleemann et al., 2009; Kobal, Van Toller, & Hummel, 1989; Porter et al., 2005; Wysocki et al., 2003). Schneider and Schmidt (1967) observed that performance in different olfactory localization tasks was the lowest for an odor possessing the lowest trigeminal qualities of the three stimuli applied in their research (coffee). Relatedly, Croy et al. (2014) found that a few people who were able to correctly lateralize PEA in their study exhibited a significantly enhanced activation of cerebral trigeminal processing areas in response to this odorant. This finding suggests that the trigeminal system of some individuals is so sensitive that it responds to odorants that most other people find to be purely olfactory, consequently increasing the lateralization abilities of this group (Croy et al., 2014). This could also explain the inconsistencies in literature, as PEA in high concentrations can also induce trigeminal perceptions (Yang et al., 2003), a notion supported by a study showing detection of PEA by two out of 15 anosmic individuals (Doty et al., 1978).

Another group of studies explored a different spatial localization ability, namely directional smelling. For example, newborns turn away from a source of an unpleasant odor (ammonium hydroxide) (Rieser, Yonas, & Wikner, 1976), which suggests that they know where it is located. Further, in an early study by Szymanski (1920), humans were able to determine the location of an odor source – an ability potentially relevant for creating spatial representations based on olfactory cues. This result was extended by Porter and collaborators (Porter et al., 2007), who showed that people are able to follow scent trails. Jacobs and collaborators (Jacobs, Arter, Cook, & Sulloway, 2015) demonstrated that it is possible to navigate to a certain location using only olfaction, and a study of location memory across modalities (Schifferstein, Smeets, & Postma, 2009) showed that people can learn to remember locations by smell to the same degree as by audition and touch. Participants in a study by Welge-Lussen, Looser, Westermann, and Hummel (2014) could also localize an odor source while sitting in a central position with quite high accuracy (85° at a 2-m distance), and this accuracy increased to approximately 40° at closer distances. Interestingly, the results of Welge-Lussen and collaborators (2014) were the same for a predominantly olfactory odorant (PEA) and predominantly trigeminal substance (cineol). Thus, even in the absence of intranasal location mediated exclusively by the trigeminal system, humans may be able to exploit differences in intensity or timing (von Békésy, 1964) to achieve relatively accurate direction smelling (spatial localization), even with purely olfactory stimuli. This is further suggested by decreased scent-tracking accuracy in a monorhinal compared to a birhinal tracking condition (Porter et al., 2007).

Correct localization of olfactory stimuli can, however, be learned (Negoias, Aszmann, Croy, & Hummel, 2013), as following olfactory training, women were better able to correctly localize a purely olfactory stimulus (PEA). Training can also improve scent tracking (Porter et al., 2007). In addition, some people are able to lateralize olfactory stimuli above chance (Croy et al., 2014; Frasnelli et al., 2010), which raises the question of what determines the spectrum of olfactory localization abilities. Possibly, some people can develop this skill based on an exposure to odors in daily situations. An example of such a group could be blind people. Some authors hypothesize that the highly developed olfactory functions observed for some blind individuals might result from daily “smell training” (Gagnon, Ismaili, Ptito, & Kupers, 2015). Here, in a series of studies we explored whether odor lateralization and localization abilities are increased in this group. Following the idea of Welge-Lüssen et al. (2014), we combined two different aspects of odor localization. First, we used the classic method used in intranasal localization tasks – in Study 1 we compared the abilities of blind and sighted groups to correctly lateralize eucalyptol, an odorant with a strong trigeminal component, presented passively to either nostril. Studies 2 and 3 involved a more ecologically valid task, namely spatial localization of an external olfactory stimulus. In Study 2 subjects tried to localize a single odorant, while in Study 3 they attempted to localize a single target odor in an experimental olfactory space comprising four different odorants.

## Materials and methods

Each study began with a short interview regarding the medical history of the subjects and any potential olfactory problems. Further, to ensure normal olfactory function, the participants completed a three-item olfactory screening test (Lötsch, Ultsch, & Hummel, 2016). In order to maintain identical testing conditions for all subjects, in all studies both blind and sighted participants were fitted with a Mindfold mask eliminating all incoming light and visual input without blocking the nose or forcing the eyes closed (Mindfold Inc., Boulder, CO, USA). All three studies took place in a quiet, large room under constant conditions. All doors and windows were always closed during testing, and no one entered or exited the room during the experiments. The room was thoroughly ventilated after each testing.

### Ethics statement

The studies were approved by the ethics board of the Institute of Psychology, University of Wroclaw. The work was performed in accordance with the Declaration of Helsinki for Medical Research involving human subjects. All participants provided written, informed consent prior to their inclusion in the study.

### Data analysis

In all studies we used IBM SPSS v. 24 software to analyze data with the alpha level set to .05.

In Study 1, for each participant we computed the proportion (percent) of correct answers by dividing it by the total number of trials taken and multiplying by 100. We compared sighted and blind individuals’ mean proportions of correct answers in a eucalyptol lateralization task utilizing an independent sample *t*-test and referred it to the expected level of chance (50%). In Studies 2 and 3, the localization performance was defined as the absolute value of a difference between the actual position of the target odorant and supposed position of the target odorant, indicated by the participants’ index finger (in degrees).

In Study 2, localization accuracy of sighted and blind individuals was compared using an independent samples t-tests. In Study 3, we investigated the spatial localization across multiple trials by means of mixed linear models (LMM) with “sightedness” (blind vs. sighted), “task” (head moves vs. no head moves), and “location of target odor” (lateral vs. central) as fixed factors, and age and ordinal number of trials as covariates. The data and applied scripts are available upon request.

## Study 1

In Study 1, we investigated subjects’ ability to lateralize eucalyptol, a mixed olfactory/trigeminal stimulus.

### Participants

Participants in this study were 69 blind (*M*_age_ = 41 ± 1.6 years; 32 females) and 45 sighted controls (*M*_age_ = 38.3 ± 2.1 years; 25 females).

### Procedure

We tested the subjects using a hand-held squeezing device (for details, see Hummel et al., 2003) consisting of two 250-ml bottles placed in a special stand. One bottle contained 15 ml of 50% eucalyptol solution (with propylene glycol used as a solvent) and one contained 15 ml of odorless propylene glycol. Each bottle had a soft, plastic spout on top – these parts were placed in the subject’s nostrils. During the testing, bottles were pressed simultaneously with the squeezing device in order to deliver an identical air puff into both of the participant’s nostrils at the same time. The participant's task was to answer in a two-alternative (left/right) forced choice paradigm the side of which had been stimulated with an odorous substance. The subjects received no feedback from the experimenter on the correctness of their evaluation. Stimulation of the left or right nostril followed a pseudorandomized sequence; the participants received between ten and 20 air puffs, and each nostril was stimulated between five and ten times. The subjects were told that they could report fatigue and withdraw from further participation in the experiment after the minimum number of ten air puffs had been reached.

### Results

**Fig. 1:**
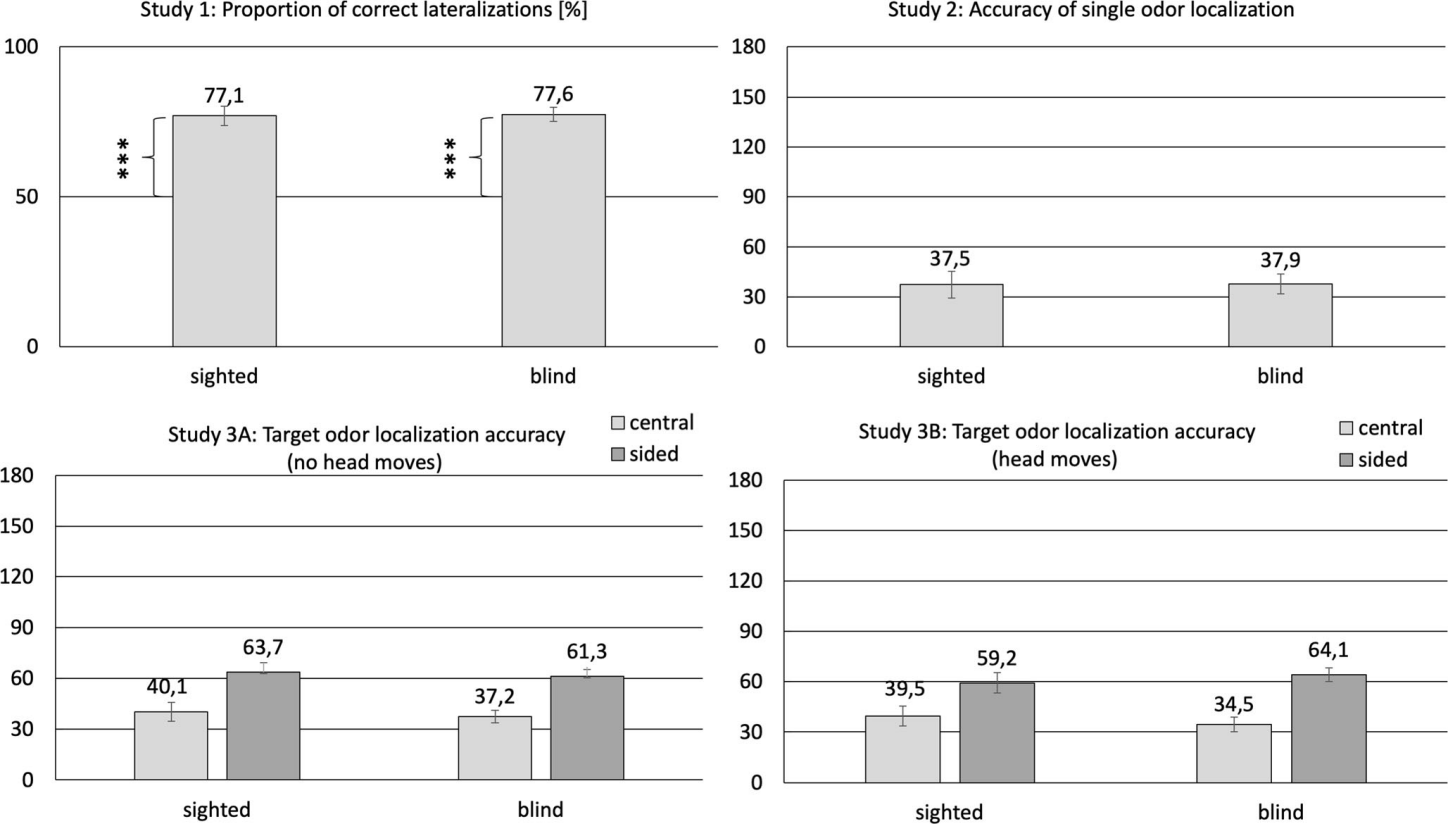
Comparison between sighted and blind individuals in terms of their accuracy in lateralization (Study 1) and localization tasks (Studies 2 and 3). In the graph presenting the results of Study 1, axis Y depicts a proportion of correct lateralizations (0–100%), whereas in the graphs illustrating the findings of the Studies 2 and 3, the scale reflects 180°, which is a maximum theoretical difference between the actual and the indicated locations of the odorous substance. Empirically, the values of this difference ranged between 0 and 160°. *** denotes a significant difference (*p*<.001) between scores obtained by the sighted and the blind individuals as compared to the expected level of chance (50%)

The results of the blind and sighted participants are illustrated in Fig. 1 (panel A). The tested model revealed no significant differences between sighted and blind participants in terms of their ability to lateralize eucalyptol, *t*(1,112)=-.13, *p*=.89. Both groups significantly exceeded the 50% level of chance when lateralizing eucalyptol. Results obtained by blind and sighted individuals were 77.6 ± 2.3 (*t*(44)=8.5, *p*<.001) and 77.1 ± 23.2 (*t*(68)=11.9, *p*<.001), respectively.

## Study 2

In Study 2, we examined subjects’ ability to localize olfactory sources in an experimental setup more similar to everyday life. The subjects were required to localize one odor that was placed on a table in front of them.

### Participants

Participants in this study were 13 blind individuals (*M*_age_ = 28.5 ± 3.5 years; seven females) and 16 sighted controls (*M*_age_ = 34.9 ± 3.2 years; ten females).

### Procedure

The method of this experiment was based on studies involving auditory (e.g., Röder et al., 1999) and olfactory localization (Welge-Lussen et al., 2014).

The participants were asked to localize the source of an odor in a 180° space in front of them. The study was conducted by two assistants – one was responsible for handling the odorants (from now on referred to as “Assistant 1”) and the second assisted the participants (“Assistant 2”). Assistant 1 placed one 50-ml bottle containing 15 ml of the odorant (orange) on a large table. Assistant 1 marked a space where the odor was placed and noted the exact angle where the odor was presented (it was supposed to be placed at approximately 0, 60, 120, or 180°). To facilitate the exact calculation of the presentation angle, a 2 x 1 m print-out scale was placed on the table.

The blindfolded participant was invited to the room and was seated by Assistant 2 on one side of the table, in the center of the print-out scale. The subject sat comfortably upright in a rotating chair with his/her head resting on an adjustable head rest and was instructed to keep the head fixed in this position. Initially, the participant was seated with his/her back turned towards the table. Assistant 2 explained the task. The participant was asked to insert earplugs after confirming that he/she understood the task. The earplugs were to be removed after the participant was turned to face the table and received a tap on the shoulder. During a few seconds when the participant was already facing the table, but his/her ears were still blocked, Assistant 1 opened the bottle containing the odorant. Ear blocking was employed in order to avoid auditory localization of the odor source during bottle opening.

Approximately 30 s after the odorant bottle was opened, Assistant 2 asked the subject to localize the odor source. The participants were strictly instructed not to move their heads during this task, and the research assistants controlled this aspect during the task performance. The subject was instructed to sniff and indicate the location of an odor source with an extended arm and index finger; Assistant 2 noted the exact angle indicated by the participant’s index finger. Task performance was calculated by subtracting the angle that a subject indicated from an angle that a smell was presented. We further analyzed the absolute value of this difference, as we did not have a hypothesis regarding the enhanced abilities on the location of the odor source to the left versus the right side of the target odor. Participants were seated approximately 50 cm from the odorant source (a distance similar to the “close distal condition” in Welge-Lussen et al., 2014).

### Results

**Fig. 2:**
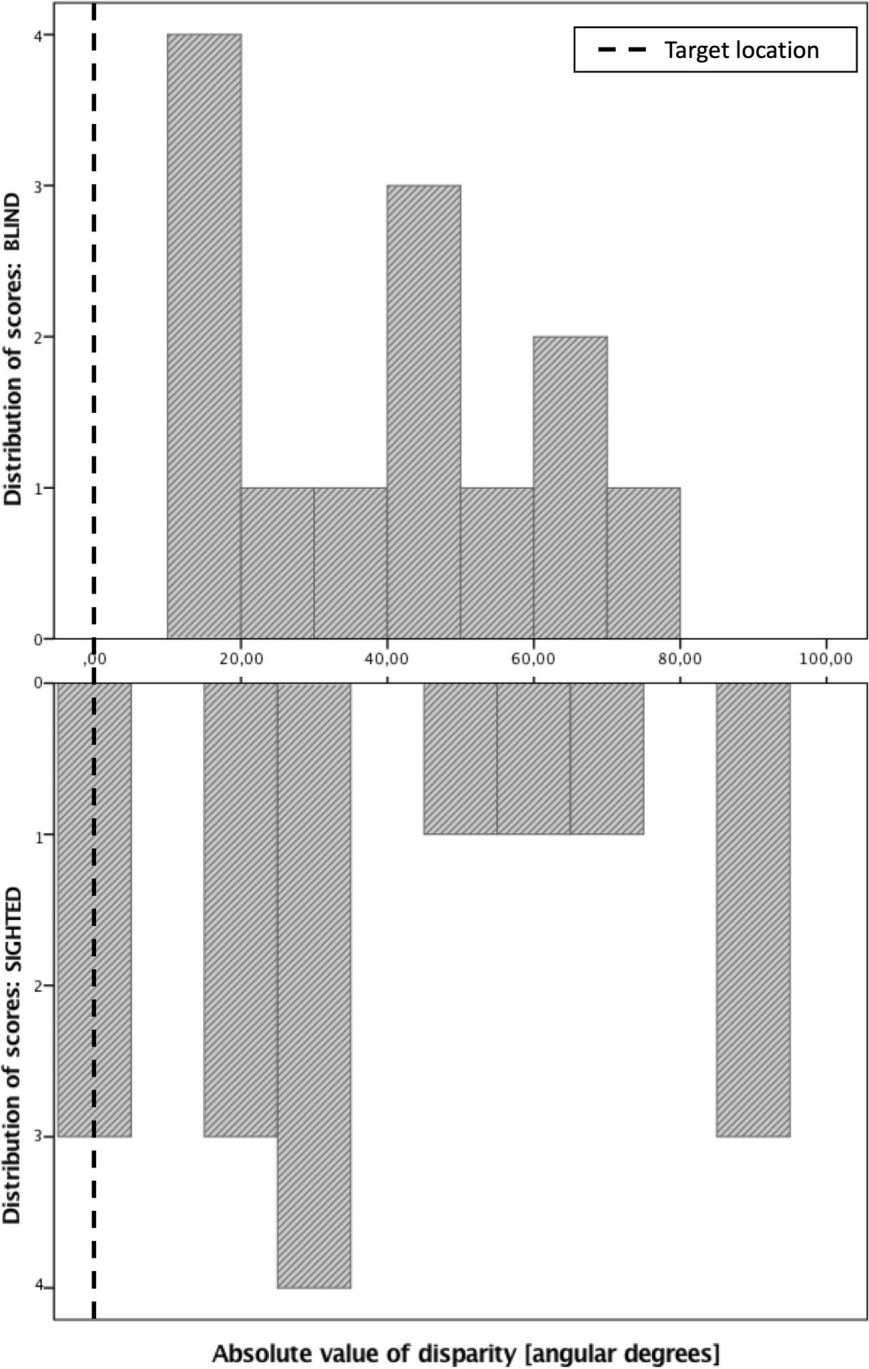
The distribution of scores obtained by the blind and the sighted participants in Study 2. The y-axis depicts the absolute value of a difference between the actual and the indicated location of the odorous substance in angular degrees

The results of the blind and sighted participants are illustrated in Fig. 1 (panel B), and Fig. 2 shows the distribution of scores of both participating groups. We found no significant difference between the localization accuracy of the blind (*M* = 37.9 ± 5.9°) and sighted participants (*M* = 37.5 ± 8.1°), *t*(27)=-.04, *p*=.97.

We further attempted to estimate the chance performance based on random pointing locations. We compared actual errors made by the participants with errors of a random distribution. We generated a variable consisting of random numbers ranging from 0 to 180 that described theoretical random indications of subjects and we subtracted these values from the actual locations of the odorants (it needs to be noted that the mean by-chance error estimates obtained in this way will vary slightly when the randomization is rerun). We used absolute values of errors of the applied random distribution and compared them with the absolute observed errors made by the subjects by means of a paired-sample t-tests. Although the difference between the mean absolute observed error (*M* = 37.7, *SD* = 27.4) and the mean absolute random error (*M* = 52.4, *SD* = 30.1) equaled almost 15°, this difference did not reach statistical significance, *t*(28)=-.18, *p*=.08. However, the number of trials for which the participants made an error below or equal to the average random error was considerable (20 out of 29), and it did not differ between the participating groups (nine trials out of 13 made by the blind subjects and 11 out of 16 made by the sighted subjects, *χ*^2^(1)=.01, *p*=.98).

## Study 3

In Study 3, the same experimental setup was applied as in Study 2. This time, however, the subjects were simultaneously presented with four smells placed at four distinct places of the experimental board (at approximately 0, 60, 120, and 180°). We selected odors that were intense, perceptually distinct and trigeminally stimulating – cinnamon, jasmine, orange, and cloves in one condition and chocolate, eucalyptol, freesia, and strawberry in the second. The participants were assigned to odor conditions randomly, and the order of substances was pseudorandomized. Every odor was dissolved in propylene glycol in order to obtain concentrations that would seem equally intense in a pretest involving smelling the solutions from a distance of 50 cm. For every odorant bottle, we prepared a corresponding Sniffin’ Stick. Assistant 2 presented the sticks to the participant one by one in a pseudorandomized order, asking him/her to indicate where the corresponding odor source was located. The participants were told that the location of the odor samples might be changed each time they pointed to a certain direction, but in reality, the positions remained the same in order to avoid odor mixing.

The participants were asked to complete two tasks. First, they were asked to localize the target odorant without moving their heads (the face was directed towards the middle of the scale and kept on a headrest; a research assistant ensured that the participants did not move). Then, they completed the same task again, but this time they were allowed to move their heads sideways.

### Participants

Participants in this study were 97 blind individuals (*M*_age_ = 43.1 ± .5 years; 48 females) and 47 sighted controls (*M*_age_ = 38.7 ± .7 years; 27 females).

### Results

**Fig. 3:**
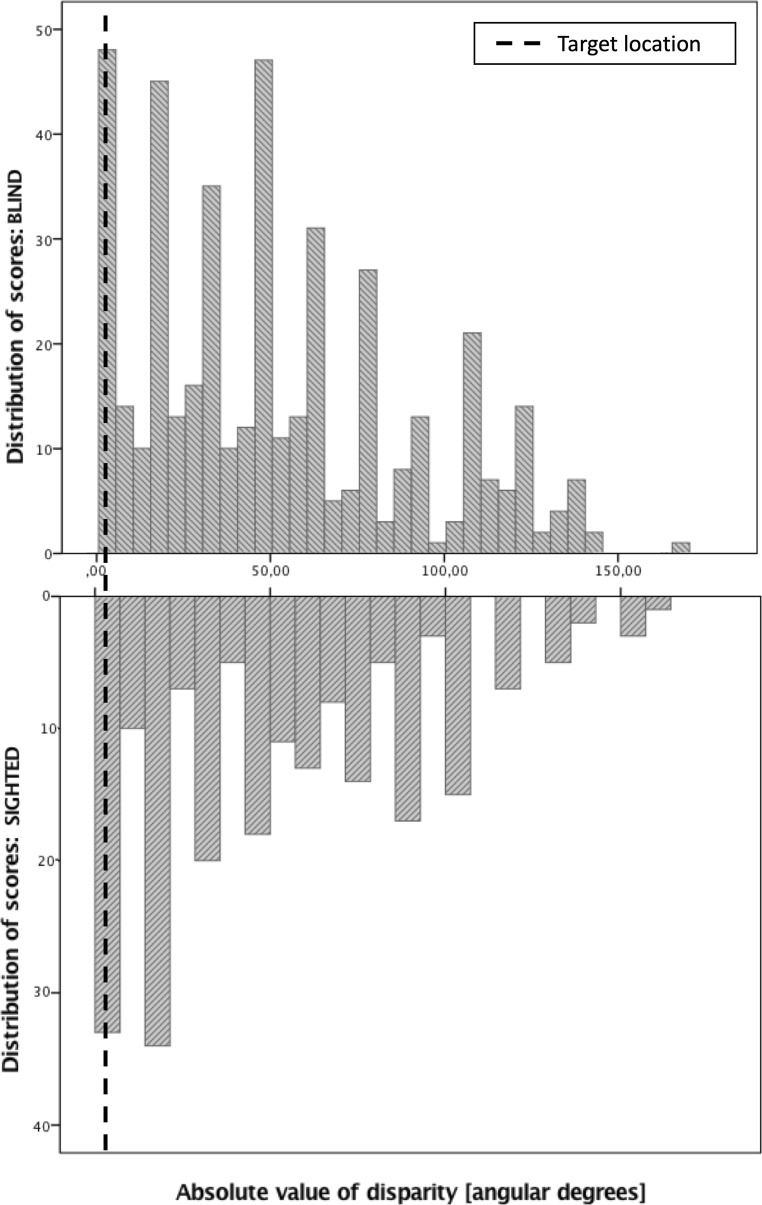
The distribution of scores obtained by the blind and the sighted individuals across two tasks in Study 3. The y-axis depicts the absolute value of a difference between the actual and the indicated location of the odorous substance in angular degrees

The results of the blind and sighted participants are illustrated in Fig. 1 (panels C and D) and Fig. 3 shows the distribution of scores of both participating groups across two tasks. General linear model with “sightedness” (blind vs. sighted), “location of target odor” (central [60 or 120°] vs. sided [0 or 180°]), “task” (no head movements vs. head movements) yielded a significant main effect of “location of target odor” in terms of accuracy in spatial localization, wherein odors located in the central positions were localized significantly more accurately (*M* = 37.8 ± 2.1°) than odors located in the sided positions (*M* = 62.1 ± 2.1°; *F*(1,675)=65.1, *p*<.001). There were no other main or interaction effects (*F*s<.75, *p*>.39).

Similar to Study 2, we further compared our subjects’ performance to the estimate of the chance performance based on random pointing locations. We found that across two tasks, the participants’ actual average error (*M*=50, *SD*=38.9) was significantly lower than the randomly generated average error (*M*=54.7, *SD*=40.3), *t*(682)=-2.4, *p*=.016. The number of trials for which the participants made an error below or equal to the average random error did not differ between the blind and the sighted groups (271 out of 452 trials made by the blind subjects and 136 out of 231 trials made by the sighted subjects, *χ*^2^(1)=.074, *p*=.79).

To further explore the significant effect of the target odor location, we estimated the chance performance based on random pointing locations separately for the central and sided locations. For sided locations, the participants’ actual average error (*M*=62.3*, SD*=43.2) did not differ significantly from the randomly generated average error (*M*=65.5*, SD*=45.7), *t*(344)=-.97, *p*=.34. The number of trials for which the participants made an error below or equal to the average random error of 65.5° when the target was on the side did not differ between the blind and the sighted groups (133 out of 234 trials made by the blind subjects and 61 out of 111 trials made by the sighted subjects, χ^2^(1)=.11, *p*=.74).

For central locations, the participants’ actual average error (*M*=37.3, *SD*=28.8) was significantly lower than the randomly generated average error (*M*=43.8, *SD*=30.3), *t*(337)=-2.77, *p*=.006. The number of trials for which the participants made an error below or equal to the average random error of 43.8° when the target was in the central position did not differ between the blind and the sighted groups (127 out of 218 trials made by the blind subjects and 64 out of 120 trials made by the sighted subjects, χ2(1)=.76, *p*=.38).

## Discussion

Our study showed that a large sample of blind subjects did not differ from sighted people in lateralization and localization of odorants. This was true for a trigeminal stimulus (eucalyptol) presented monorhinally and various odors presented birhinally in three different conditions. The level of performance of both groups across all tasks suggests that odor lateralization and localization are very important regardless of visual status, most probably because these abilities can protect the respiratory tract and aid navigation in an environment.

We observed no differences between the blind and the sighted participants in odor lateralization. Lateralization abilities correlate with overall olfactory sensitivity (Hummel et al., 2003). It seems then that this aspect of olfactory function is yet another element of sensory perception where no compensation is observed in blindness. However, it should be noted that both blind and sighted participants scored significantly above the level of chance when lateralizing eucalyptol. This finding is consistent with previous works showing that trigeminally stimulating substances can be accurately lateralized intranasally (Frasnelli et al., 2010; Hummel et al., 2003; Kleemann et al., 2009; Kobal et al., 1989; Oleszkiewicz et al., 2017; Porter et al., 2005; Wysocki et al., 2003). As trigeminal stimuli are usually associated with burning, tickling, stinging, and even painful sensations (Doty et al., 1978), an evolutionary point of view suggests there is an advantage in precisely localizing the source of these threats (e.g., environmental hazards). This notion is further confirmed by studies showing that somatosensory sensitivity to trigeminal stimuli is higher in the anterior than in the posterior part of the nasal cavity (Scheibe, Schmidt, & Hummel, 2012), which suggests that the trigeminal system could protect the olfactory system (and the entire respiratory tract) from toxic stimuli. Further, habituation effects are much lower for trigeminal than for olfactory stimuli (Sinding et al., 2017), so even repeated (like in our experiment) or prolonged exposure to such stimuli should not affect perception accuracy. In the case of our paradigm it is also important that intensity of trigeminal stimulation is associated with the accuracy of odor localization (Frasnelli, Hummel, Berg, Huang, & Doty, 2011; Frasnelli & Hummel, 2005; Hummel et al., 2003; Schneider & Schmidt, 1967). Dangers associated with trigeminal stimuli can be detected independent from visual input, and because of a direct pathway between the olfactory receptors and the human brain (Stockhorst & Pietrowsky, 2004), accurate reactions can be possible without the mediating effect of sight. No compensatory effect of blindness in this case might thus result from equally high importance and processing abilities of trigeminal stimuli for blind and sighted people. It would, however, be of interest to compare results when using a pure odor with the findings on trigeminal stimuli. Given that olfactory stimuli do not need to be associated with avoidance of hazards (beneficial both to blind and sighted people), the potential compensation-driven differences depending on the visual status might be more pronounced in the case of pure olfactory stimuli.

In a series of experiments, we observed no differences in odor localization between blind and sighted participants. Both groups were able to localize applied odors with quite high accuracy. In a task involving only one odor, the accuracy reached a mean level of 37 (Study 2), and for four odors this was 50° (Study 3), a result very similar to that of Welge-Lussen and collaborators (Welge-Lussen et al., 2014), where localization accuracy in a near-field condition (a distance of 40 cm) ranged between 36 and 47°. This means that although our subjects were not able to precisely point to the source of odor, they were capable of indicating a rough direction where it was coming from. It should also be mentioned that in Study 3, we compared localization abilities for “central” (60°, 120°) and “lateral” (0°, 180°) odor sources. We assumed it would be easier to roughly distinguish the side of stimulation when the olfactory stimulus was placed laterally than to decide if it is placed more on the left or right when it was in front of a subject. This would also be expected following the line of thinking presented by Von Békésy (1964), and previous findings in the area of auditory processing in the blind people (Röder et al., 1999). However, quite contrary to our hypothesis, we found significant differences in localization accuracy for these two conditions, wherein odors located in the central positions were localized significantly more accurately than odors located in the sided positions (the performance of the blind and the sighted people was still similar in these two cases). This finding is also very intriguing from an evolutionary perspective. We could assume that it is more useful to precisely distinguish the lateral locations of olfactory stimulation, as olfactory perception could in such a case supplement information about, for example, potential dangers present in the far peripheral visual field.

It is very interesting that there seemed to be no transfer of sensory function for odor localization in most of the blind people. Our results are consistent with numerous studies regarding null effects of visual impairment on performance in various olfactory tasks (e.g., Cornell Kärnekull, Arshamian, Nilsson, & Larsson, 2016; Guducu, Oniz, Ikiz, & Ozgoren, 2016; Luers et al., 2014; Majchrzak, Eberhard, Kalaus, & Wagner, 2017; Smith, Doty, Burlingame, & McKeown, 1993; Sorokowska, 2016; for a recent review and meta-analysis on this issue, see Sorokowska, Sorokowski, Karwowski, Larsson, & Hummel, 2019). This pattern of findings suggests that the effect of blindness on olfactory functions is not a simple reaction of the organism to a sensory loss (for a review see: Kupers & Ptito, 2014). Nevertheless, some blind individuals do have a better sense of smell than sighted people (Rombaux et al., 2010). Possibly there exist some other factors, other than visual impairment itself, that affect potential enhancement of olfactory processing. We have a few hypotheses as to why we did not observe any superiority of blind people specifically in odor-localization tasks. First, blind people could rely on audition and touch to a greater extent than on olfaction in the case of motor skills and spatial orientation, especially because sensory compensation for audition and touch is observed more consistently than for olfaction (Kupers & Ptito, 2014). Additionally, materials and aids for blind and visually impaired pupils and students are mostly based on tactile and auditory stimulation. Even if the brains of blind people are reorganized and olfaction of blind people is theoretically better than that of the sighted individuals (Kupers et al., 2011; Renier et al., 2013; Rombaux et al., 2010), reliance on auditory and tactile sources of information might diminish the relative importance of olfaction in navigation. Indeed, tactile cues are often used for blind people’s spatial orientation (Leonard & Newman, 1970), and although odors might be used for localization, the olfactory system is generally a poorer localizer than vision and hearing (Jacobs et al., 2015). Some authors suggest that, contrary to vision and audition, olfaction is a sense used primarily for proximate stimuli, and it might play a less important role in spatial orientation based on distal stimuli (Köster, 2002).

We could also hypothesize that our null findings were due to the experimental designs that were not sensitive enough to capture the effect of sensory compensation. However, it should be noted that the localization abilities have never been tested before in the context of olfactory compensation, and that similar studies in the area of auditory processing showed enhanced localization skills in blind individuals (Lessard, Paré, Lepore, & Lassonde, 1998; Röder et al., 1999). The tasks completed by our participants were not easy. Performance of our subjects in the studies that were designed especially for the purpose of this research (Studies 2 and 3) was similar in the blind and sighted groups, but it was still quite low. There was no ceiling effect in our samples, and should the compensatory-driven enhancement of odor localization abilities be observed, the blind subjects could easily perform much better.

Finally, it is also possible that although we intended our spatial localization studies to be as ecologically valid as possible, focusing selectively on odors might not have been very natural for our subjects. A general property of navigation is that locations are encoded redundantly, often using more than one sensory system (Jacobs et al., 2015). Further, we focused the attention of the participants on olfactory stimulation, regardless of their visual status. In a wayfinding experiment of Passini and Proulx (1988), the authors showed that, compared with sighted individuals, totally blind people noticed and processed more information (including olfactory cues) as landmarks during wayfinding. Future studies could focus on two different aspects – first, whether sensory compensation in blindness is found in tasks involving cross-modal processing, and second, whether sighted and blind individuals also perform equally well in tasks where their attention is not directed towards olfactory information.
